# Langat Virus Infection Can Be Demonstrated in Both Tick Salivary Glands and Midgut Within 24 Hours of Blood Feeding

**DOI:** 10.3390/v18050505

**Published:** 2026-04-28

**Authors:** Missiani Ochwoto, Danielle K. Offerdahl, Edwin O. Ogola, Barbara C. Weck, Dan Long, Greg A. Saturday, Marshall E. Bloom

**Affiliations:** 1 Biology of Vector Borne Viruses Section, Laboratory of Virology, Rocky Mountain Laboratories, Division of Intramural Research, National Institute of Allergy and Infectious Diseases, National Institutes of Health, Hamilton, MT 59840, USA; 2 Rocky Mountain Veterinary Branch, Rocky Mountain Laboratories, Division of Intramural Research, National Institute of Allergy and Infectious Diseases, National Institutes of Health, Hamilton, MT 59840, USA

**Keywords:** tick-borne viruses, flaviviruses, ixodid ticks, *Ixodes scapularis* ticks, Langat virus

## Abstract

The detailed mechanism and sequence by which tick-borne flaviviruses (TBFVs), such as Langat virus (LGTV), infect and disseminate in arthropod hosts remain undefined. To begin characterizing these processes, we used artificial membrane feeding chambers to feed adult Ixodes scapularis ticks with blood containing LGTV. At 24, 48, 72, and 96 hours (h) after attachment, we removed and dissected the partially fed ticks to obtain the midgut and salivary glands. Histology confirmed infection in cells of the digestive epithelium lineage; infection was noted in midgut generative cells and the more differentiated functional digestive cells over the course of feeding. The viral envelope (E) protein, nonstructural protein 3 (NS3), and double-stranded RNA (dsRNA) were readily detected in these cells by 48 h after infection. Parallel analysis indicated that cells in salivary gland acini were also infected by 48 h, where virus target cells appeared to be the granular cells in acini types II and III. Thus, both salivary glands and midgut showed direct evidence of infection by 48 h. Although viral staining was not observed at 24 h, when organs were removed at 24 h and individually cultured ex vivo, the virus was detected. Taken together, our results provide evidence of LGTV infection in both the salivary glands and midgut within the first 24 h of a blood meal. The findings should prompt a reevaluation of the systemic dissemination of TBFV in infected ticks.

## 1. Introduction

Infections with tick-borne flaviviruses (TBFVs) represent a burgeoning worldwide public health problem [1]. In 2020, there were an estimated 3734 cases of tick-borne encephalitis virus (TBEV) infections reported, 125% higher than the 2015 reported cases in Europe [2,3,4], despite an effective vaccine [4,5]. In addition, in the United States, infections with the autochthonous Biosafety Level 3 (BSL3) Powassan/deer tick virus (POWV/DTV) are also on the rise, although the overall numbers are relatively low [6]. TBFV infection is often asymptomatic but may be associated with chronic debilitating neurological signs and even death [5,6].

In nature, TBFVs are maintained through a transmission cycle involving an ixodid tick vector and various vertebrate hosts [7]. In North America, the primary vector for TBFV is *Ixodes scapularis*, and in Europe, it is *Ixodes ricinus* [8,9,10]. Virus infection in the tick is persistent and maintained both transstadially and transovarially [11]. Like other ticks, *I. scapularis* is an obligate hematophagous arthropod that feeds on different hosts at each developmental stage [11,12]. Transmission of the virus to vertebrate hosts is extremely rapid, as short as 15 min in some studies [13,14,15], and ticks can horizontally transmit the virus to other ticks via co-feeding [11]. Humans are inadvertent hosts [16]. TBFV Langat virus (LGTV) can serve as a convenient model for studying many aspects of POWV/DTV infection under BLS2 laboratory conditions.

Despite the critical role that infected ticks play in TBFV infection, the precise biology of infection in the arthropod hosts has been understudied. *I. scapularis* exhibits prolonged feeding of up to 10 days for the adult female [17,18]. It is during blood meals that a tick acquires the virus from infected animals [19,20]. In very general terms, it is believed that the virus moves through the esophagus to the midgut lumen, whence it is disseminated throughout the tick [21], and after molting, the virus is transmitted to the next host via the tick’s infected saliva during the blood-feeding process [20]. TBFV replicates in the epithelial cells of the digestive tract in nymphs and adult ticks [7,22], as well as in the salivary glands of infected *I. scapularis* ticks [23,24]. Nevertheless, the detailed biology of tick–virus–host interactions and the sequence in which tick organs are infected in arthropod hosts are not well studied [23].

The present study utilized an artificial membrane-based tick feeding system to provide a detailed description of LGTV infection in adult *I. scapularis* ticks. Looking specifically at the midgut and salivary glands, we determined that midgut digestive cells and salivary gland granular acini are likely primary targets of TBFV infection. Evidence of infection in both organs was detected within 24 h of feeding and increased over time.

## 2. Materials and Methods

### 2.1. Obtaining and Maintaining Ticks

All ticks and tick frass used in this study were purchased from the Oklahoma State University tick-rearing facility (Stillwater, OK, USA). The ticks were maintained in the lab at 25 °C, 90% humidity, and a regulated light/dark cycle period.

### 2.2. Artificial Membrane Feeding and Infection

We used the artificial membrane chamber protocol as described by Oliver et al. with slight modifications [25]. Briefly, equal volumes of silicone components A and B of Eco Flex 00-05 (Ecoflex Supersoft 00-50, Smooth-On Inc., Macungie, PA, USA) were combined with hexane (final concentration of 20%) and mixed thoroughly. The silicone paste was poured onto 4 × 6 inch lens paper (Fisher Scientific, Pittsburgh, PA, USA) taped to a plastic transparency sheet and thoroughly rubbed with gloved fingers untill the membrane had an even wet look. The excess silicone was scraped off to obtain a membrane with a target thickness of 60–100 µm, verified using a micrometer. The wet membrane was allowed to cure overnight. To attach feeding chambers to the membrane, 5–10 mL part A component of Mold Max 30 (Mold Star 30, Smooth-On Inc., Macungie, PA, USA) was poured into a 50 mL conical tube. Using graduated marks on the tube to estimate volume, 1/10 of the volume of the part B component of Mold Max 30 (Mold Star 30, Smooth-On Inc., Macungie, PA, USA) was added. The mixture was stirred thoroughly to mix, then scraped into a weighing boat. The chamber was lightly dipped into the silicone and twisted slightly to ensure a complete seal before being placed on the membrane sheet. The silicon was allowed to cure overnight, then the chambers were cut and trimmed. The integrity of the chambers was tested by adding 5 mL of 70% ethanol; chambers that leaked were discarded.

### 2.3. Feeding and Infecting Ticks with Langat Virus (TP21)

A liquid frass extract was prepared by soaking 100 mg/mL raw frass in water overnight, removing solid material by centrifugation, and adding 1 mM tripeptide reduced glutathione before filter sterilization. Immediately prior to use, 10 µL of liquid frass extract was added to the membrane. After drying for one hour, about 30 pellets of raw solid frass and several strands of deer or elk hair were added (the deer or elk hair was obtained from deer and elk skins we purchased locally and stored at room temperature in a dry place in the laboratory). For the actual blood feeding, a maximum of 20 adult ticks with equal numbers of males and females were added to each feeding chamber, and the top was covered with a piece of mosquito net to prevent ticks from escaping [26]. The chambers were floated on 4 mL of sterile defibrinated bovine blood (Hemostat Laboratories, Dixon, CA, USA) supplemented with 2 mg/mL of glucose, 1 mM of adenosine triphosphate (ATP) and 1 mM of glutathione in wells of a 6-well plate and incubated at 37 °C.

For virus infection, 7.5 × 10^3^ ffu Langat virus (TP21)/mL of blood was added to each well. Every 10–15 h, the chambers were removed from the old well; the membrane bottom was rinsed with 10–20 mL of 1× phosphate-buffered saline (PBS) (Life Technologies, USA) and then placed in a new 6-well chamber with fresh blood and virus (for infected wells), for further incubation at 37 °C. We carefully checked the attachment process at every blood change and recorded it with photos. Caution was taken to not disturb the process, and by 36 h, all unattached ticks (both male and female) were removed from the chambers. At 24, 48, 72 or 96 h after attachment, the feeding ticks were removed from the chambers and subjected to dissection immediately or held for 72 h before dissection.

### 2.4. Tick Dissection

One subset of the removed ticks was dissected within an hour after removal. The ticks were first sterilized and cleaned to remove blood or frass by dipping them for 15 s in 70% ethanol then in 30% hydrogen peroxide. The ticks were chilled on ice before dissection as previously described [27]. The salivary glands and midgut from each tick were separately placed into wells of an 8-wells Nunc Lab-tek dish chamber slides (Cat #154941, ThermoFisher, Ashville, NC, USA) with 300 µL L15C-300 medium (Gibco, Waltham, MA, USA) and used as previously described [22].

Another set of the removed ticks was cleaned, sterilized and dissected. Midgut and salivary glands organs were removed and cultured ex vivo for an additional 72 or 168 h as described [22]. The intent was to allow time for incipient infection to manifest.

### 2.5. Histopathological Assays

To evaluate the morphological changes in infected and uninfected *I. scapularis* ticks, two sets of samples were prepared for histology. The first consisted of undissected ticks removed after feeding for 48 h, 72 h, 96 h, and 144 h and at repletion (between 192 and 240 h). The second set comprised dissected tick midgut and salivary glands collected at the same timepoints.

The legs and the head of undissected whole ticks were removed to allow Bouin’s fixative and other reagents to penetrate internal organs. The undissected ticks and the dissected organs were fixed in Bouin’s solution (Cancer Diagnostic, Durham, NC, USA) for a minimum of 24 h [28]. The samples were next dehydrated in a graded series of ethanols (70%, 90%, twice in 95% for 1 h each, and 3 times in 100% ethanol for 45 min), followed by clearing in xylene for 45 min. Wax infiltration was performed at 60 °C for one 30 min cycle and three additional 45 min cycles. This was followed by embedment in pureAffin paraffin polymer (Cancer Diagnostic). For salivary glands, an additional set of unfed salivary glands samples was prepared for comparison.

Cross-sectional or longitudinal sections of the paraffin-embedded samples were cut at 5 μm thicknesses. The sections were mounted on microscope slides and stained with standard hematoxylin and eosin (H&E) or periodic acid–Schiff (PAS) staining. Images were captured by an Olympus DP70 digital camera(Olympus Corporation, Tokyo, Japan) at the magnification indicated on each image.

### 2.6. Immunohistochemical Staining

For immunohistochemical staining, the dissected organs were placed in a Labtek dish with 300 µL L15C-300 medium. The organs were washed thrice with PBS before incubation in 300 µL of 4% formaldehyde in PBS for 20 min at room temperature (RT) with rocking. Then, 300 µL of 0.1% Triton X-100 in PBS was added and incubation proceeded for an additional 20 min at RT with rocking. The samples were rinsed twice with Dulbecco’s phosphate-buffered saline (DPBS) (ThermoFisher Scientific, Waltham, MA, USA), and free aldehydes were quenched in 50 mM of glycine in DPBS at RT for 5 min while rocking. After 3 washes with DPBS, 2% BSA in PBS was added, and the mixture was incubated for 60 min at 37 °C in 5% CO_2_ with rocking.

Sections were incubated in primary antibody directed against dsRNA and NS3, and E proteins [22] were added (dilution 1:500) in separate wells. The primary antibody was incubated at 37 °C with 5% CO_2_ with rocking for 12 h or overnight. Sections were washed three times for 5 min with DPBS prior to incubation for 2 h in a secondary antibody (dilution 1:500) at 37 °C with 5%, CO_2_ with rocking, protected from light. The secondary antibodies were either anti-mouse or anti-chicken specific IgG conjugated with Alexa Fluor 488 or Alexa Fluor 594 (Life Technologies, USA). The organs were washed three times for 5 min with DPBS. Cell nuclei were stained with 1:100 dilution of Hoechst NucBlue stain (Life Technologies-Invitrogen) and polymerized actin in smooth muscle cells was labeled with Alexa Fluor 488 phalloidin (Life Technologies) and incubated for 60 min. After three final washes in DPBS, the sections were transferred to a 0.15 mm microscope cover glass (Fisher Scientific, Pittsburgh, PA, USA) and mounted in Prolong Gold Antifade (Life Technologies-Invitrogen, Eugene, OR, USA) on a 1 mm thick microscope glass slide.

The specimens were observed and images captured on a Zeiss LSM 710 confocal microscope (Carl Zeiss Microscopy GmbH, Jena, Germany) driven by ZEN Black v2.3 software.

### 2.7. Confirmation of Virus Infection

To confirm that the organs were infected, both infected and uninfected ticks were dissected as described above. The midgut and salivary glands were washed three times in PBS and then transferred into wells of a 8-well Labtek dish with 300 µL L15C-300 medium [29]. The organs were cultured, and the supernatant was collected and replaced with fresh medium after every 24 h for eight days as previously described [22].

Virus titration of the collected supernatant was performed using an immunofocus assay. Briefly, 1.0 × 10^5^ Vero cells were seeded in complete Dulbecco’s modified eagle medium (DMEM) and plated in a 12-well plate and incubated overnight at 37 °C with 5% CO_2_. Ten-fold dilutions of virus were prepared in DMEM, and 0.25 mL of each dilution was added in duplicate to the cells. After one hour of incubation at 37 °C with 5% CO_2_, cells were washed with DPBS, and 1 mL methylcellulose was added. Samples were incubated at 37 °C in 5% CO_2_ for 3 days. The immunofocus assay was developed as described [29].

## 3. Results

### 3.1. Tick Feeding Behavior

**Figure 1:**
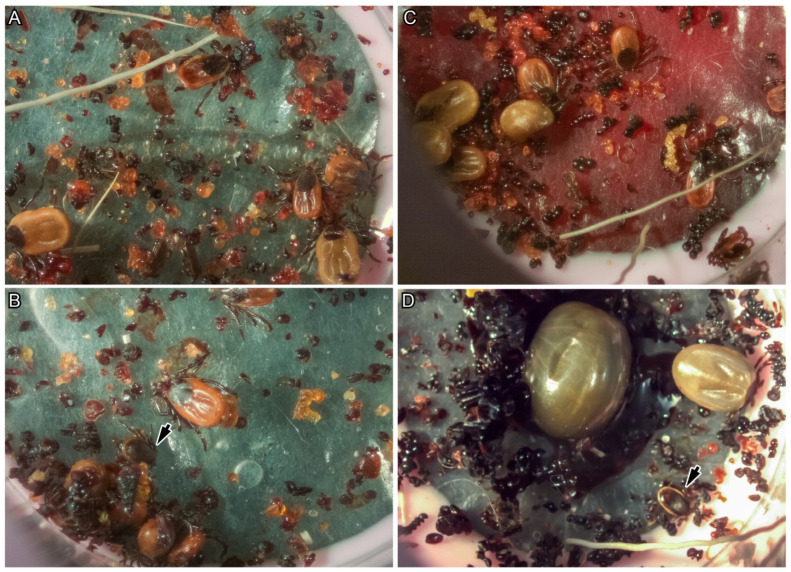
Tick feeding process using artificial membrane chambers. (**A**) Tick feeding process after removing the deer hair. (**B**) The ticks were seen feeding singly in the chambers as compared to (**C**), where most ticks fed in clusters. A few males (indicated by arrows) attached and fed for very short periods; they did not feed to engorgement compared to the females in the same feeding chambers. (**B**,**D**) Males fed with females in clusters or independently. Female ticks fed at different rates in the same chamber (**D**). Female ticks continuously fed to engorgement, but males did not.

The use of artificial membrane-based chambers proved [26] to be a suitable method for feeding adult ticks on virus-infected or uninfected blood. Attachment to the membrane occurred as early as 12 h after exposure to the blood and at 36 h the elk hair and any unattached ticks were then removed. The attachment rate was between 30% and 40% of the ticks in the membrane chambers. The ticks preferred to feed at the chamber periphery (Figure 1A–C) and fed either singly (Figure 1A) or in clusters (Figure 1B,C). There was no discernible difference observed in feeding behavior (in numbers attached, time taken to attach, and the increase in size) between ticks in the infected or uninfected chambers.

**Figure 2:**
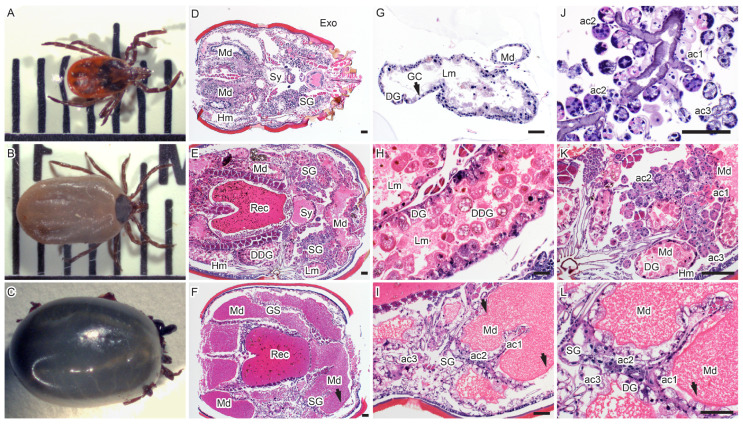
Effect of blood feeding on tick midgut and salivary glands. Morphological changes in fed *I. scapularis* tick. (**A**,**D**,**G**,**J**) Images of an unfed tick. (**B**,**E**,**H**,**K**) Images of partially fed tick, removed after 72 h of feeding. (**C**,**F**,**I**,**L**) Fully fed tick after 264 h of feeding. (**A**) An unfed tick on a 1 mm scale measures about 3 mm; the scutum is clearly visible. (**B**) After 72 h of partial feeding, the tick measured about 5 mm, and the scutum was partially visible. (**C**) When fully fed, the tick increases in size and width with the scutum barely visible. (**D**) Histological section stained with H&E showing the internal organs of an unfed tick. (**E**) The midgut and rectal sac enlarge during feeding. (**F**) As feeding progresses, enlargement of the midgut and rectal sac displaces other internal organs. (**G**) The midgut of an unfed tick has one single layer of generative cells; as the tick feeds, the number of layers increases. (**H**) Midgut of a partially fed tick has three layers of cells. The generative cells mature into digestive cells, which later detach into the lumen. (**I**) The midgut of fully fed ticks has mostly digestive cells; the cell membrane is stretched thin (also Appendix A). (**J**) Grape-like structure of the salivary glands of unfed ticks, with the three different acini clearly identified; acini type I (ac1) is not granulated, while acini type II (ac2) and type III (ac3) are granulated. (**K**) Salivary glands of a partially fed tick; all three different types of acini are identified. The midgut is in very close proximity to the salivary glands. (**L**) The salivary glands have a single layer with an enlarged lumen (Appendix A). Key: Exo = exoskeleton, Md = midgut, Sy = synganglion, SG = salivary glands, Lm = lumen, GC = generative cells, DG = digestive cells, DDG = detached digestive cells, Hm = hemocoel, Rec = rectal sac, ac1 = acini type 1, ac2 = acini type 2, ac3 = acini type 3, The arrows in (**F**,**I**,**L**) point to the midgut membrane. The bar represents 100 µM.

During the slow feeding phase (between attachment and 120 h), the female ticks increased in size by approximately a millimeter in length in 24 h increments up to about 5–6 mm (Figure 2A,B). During the fast feeding phase (between 120 h and 192 h), the female ticks maintained the 5–6 mm length but increased in width and girth (Figure 2B,C). Occasionally, the males were attached to the female genital pore even after removing the female from the membrane. In addition, we observed a few males attached briefly to the membrane that would feed for less than six hours, as shown by the arrow in Figure 1B,D; we did not observe any male feeding continuously to engorgement.

### 3.2. Effect of Blood Feeding on Tick Midgut and Salivary Glands

We next characterized the effects of blood feeding on the midgut and salivary glands. Examination of paraffin-embedded sections stained with hematoxylin and eosin (H&E), revealed that the tick midgut and rectal sac increased tremendously in size during feeding (Figure 2D–I).

The midgut wall of unfed ticks comprised a thin layer of generative cells (GCs) separated from the circumferential network of smooth muscle cells by a basement membrane (Figure 2G). During the slow feeding phase, the GCs proliferated and differentiated into mature digestive cells (DGs) and many detached digestive cells (DDGs) were observed in the lumen by 72 h of feeding (Figure 2E,H).

The tick salivary glands also undergo pronounced changes during a blood meal. The salivary glands increase in size as the ticks feed (Figure 2F,I,L); however, in fully fed ticks, the salivary glands reduce greatly in size. The three types of salivary gland acini, type I (ac1), type II (ac2) and type III (ac3), were discernible in H&E sections (Figure 2J–L).

**Figure 3:**
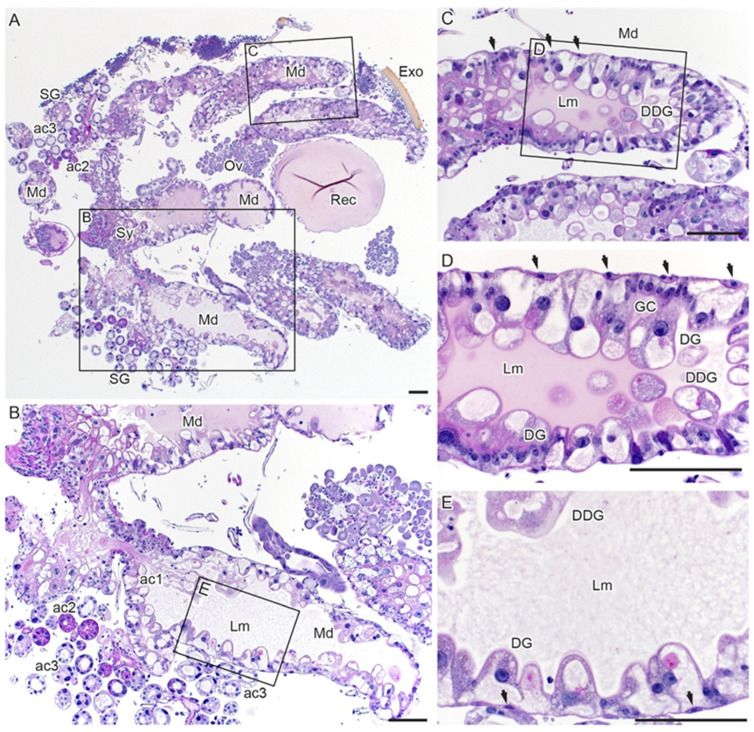
Histological sections of partially fed *I. scapularis* stained with periodic acid–Schiff (PAS). The tick was fed for 48 h and was held for 72 h before dissection. PAS stain mucosubstances, primarily the basement membrane (**A**) internal organs of a tick with the back removed, showing midgut diverticula and different salivary gland acini. (**B**) Higher-magnification section of panel (**A**) showing different types of acini and midgut generative cells differentiating to digestive cells. (**C**–**E**) Higher-magnification images of the insert shown in panel (**B**), showing midgut generative cells differentiating into digestive cells and detached digestive cells. The arrows point to the midgut muscle cells. Key: GC = generative cell, Exo = exoskeleton, Md = midgut, Rec = rectal sac, SG = salivary glands, Lm = lumen, DG = digestive cells, DDG = detached digestive cells, ac1 = acini type 1, ac2 = acini type 2, ac3 = acini type 3, Ov = ovary, Sy = synganglion. The bar represents 100 µM.

To better visualize these changes, we utilized periodic acid–Schiff (PAS) staining of partially fed tick midguts to stain the glycan-rich basement membrane and mucosubstances (Figure 3A–E). The simple epithelial monolayer of GC changed from cuboidal to DG with columnar morphology, increasing both in size and quantity (Figure 3A,B). The DG of fed ticks detached into the lumen, and in some sections of the midgut, we observed three layers of cells: the GC, young DG and DDG (Figure 3D). The DDGs are spherical and granulated and were observed detached in the intestinal lumen (Figure 3D compared to Figure 2H). They increased in number during tick feeding, but we noted that they were absent in the fully engorged ticks (Figure 2E,F and Figure 3C,D). The number of smooth muscle cells did not increase in size compared to digestive cells as pointed out in Figure 3C–E (arrows). When the ticks were allowed to feed until repletion (192 h), very few GCs or DGs remained attached to the basement membrane, which appeared overstretched and very thin (Figure 2I). In one instance, only the basement membrane and muscle cells were observed (Figure 2F,I).

**Figure 4:**
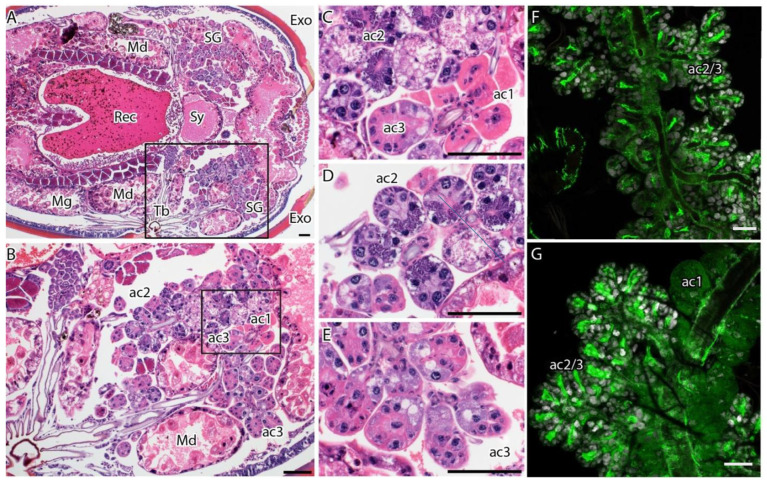
Effect of blood feeding on salivary glands of female adult *I. scapularis* ticks. (**A**–**E**) Histological sections stained with H&E, whereas (**F**,**G**) are immunofluorescent stains of the salivary glands. (**A**) A section of a whole tick showing the location of the salivary glands. (**B**) Location of the salivary glands: while feeding, the salivary glands and the midgut are close to each other. (**C**) Cytoarchitecture of acini type I, non-granulated, with a homogeneous cytoplasm that is slightly stained by eosin. (**D**,**E**) Type II and III acini showing the dense granules at the lobular duct regions of the acini. (**F**,**G**) Granules of acini II and III could not be noticed in immunofluorescence stains. Polymerized actin clearly stains the duct in acini I (compared to panel (**C**)) and distinguishes it from acini II and III (ducts are stained green and nuclei are stained blue). Key: GC = generative cell, Exo = exoskeleton, Md = midgut, Rec = rectal sac, SG = salivary glands, Mg = midgut lumen, Tb = tubules (tracheae), ac1 = acini type 1, ac2 = acini type 2, ac3 = acini type 3. The bar represents 100 µM.

When the salivary glands are stained with periodic acid–Schiff (PAS) (Figure 3A,B), type I acini (ac1) do not contain granules and are along the main salivary duct; they are largely spherical, and the acinar cells feature a homogeneous, slightly eosinophilic cytoplasm (Figure 2J,K and Figure 4B,C). Type II and III acini attach to lobular ducts, and granules of the type II acinar cells are more strongly stained in PAS or eosin-dense (in H&E sections) as compared to type III acinar cells (Figure 3B and Figure 4D,E). Within the acini, cells proximal to the lobular duct had more granules than cells more distally from the duct (Figure 3B and Figure 4D,E). The granules of acini II and III were not evident in immunofluorescence stains. Polymerized actin in muscle cells stained with phalloidin could only be used to identify acini I (Figure 4F,G) from acini II or III (ac2/3) and could not distinguish acini II from acini III (Figure 4F).

As the female ticks feed, both acini II and III increase in size and shape. In a fully fed tick, acini II and III are spherical with a large lumen (Figure 2K,L). The enlarging midgut expands, bringing it into close proximity with the expanding salivary glands due to limited space within the body cavity (Figure 2K,L and Figure 4A,B).

### 3.3. Characterization of LGTV Infection in the Midgut of Blood-Feeding I. scapularis Ticks

**Figure 5:**
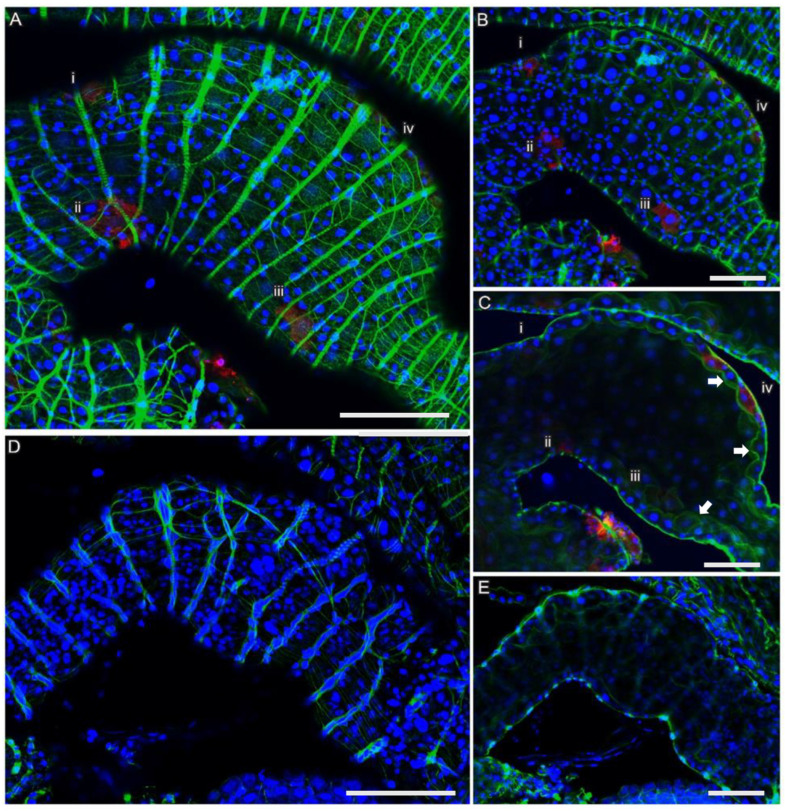
LGTV infection in the midgut of 48 h blood-feeding *I. scapularis* ticks. Images of confocal z-series microscopy of infected (**A**–**C**) and uninfected (**D**,**E**) sections of midgut diverticular with special emphasis on cells located at positions i, ii, iii, and iv. (**A**) The muscle network (stained with phalloidin, green) in the outermost section of the diverticula, which contained polymerized actin (phalloidin), was uninfected. (**B**,**C**) Infected digestive cells at positions i, ii, iii, and iv that were located deeper are clearly visible in the z-series. (**B**) Inner lining of digestive cells has polymerized actin (iv) that was due to an increase in microvilli (pointed to by the arrows). (**D**,**E**) Images of a confocal z-series microscopy of an uninfected fed tick midgut. The midgut was stained for polymerized actin (phalloidin, green), LGTV NS3 protein (red), and nuclei with Hoechst NucBlue (blue). The arrows point to the brush borders or microvilli [30]. The bar represents 100 µM.

Having characterized the effects of blood feeding, we sought to determine how virus infection would be manifest in the infected midgut. Adult *I. scapularis* ticks were fed on blood containing LGTV using the artificial membrane system detailed above. At the indicated times, ticks were removed and dissected. The location of virus infection in the midgut was identified by immunohistochemistry. When sections were stained for NS3 and E protein, direct evidence of viral infection was observed in the midgut of ticks fed for 48 h (Figure 5). Based on morphology and location, the infected cells were GC and DG (Figure 5A–C). The infected cells were solitary at early timepoints (Figure 5A–C) but formed clusters at 72 h (Appendix B). There was no evidence of viral infection in cells of the smooth muscle cell network, as shown by phalloidin staining (Figure 5A–C and Appendix B). Of interest, the brush borders or microvilli of the digestive cells of fed ticks increased in size and stained positive for polymerized actin with phalloidin [30] (Figure 5C).

### 3.4. Characterization of LGTV Infection in the Salivary Glands of Blood-Feeding I. scapularis Ticks

**Figure 6:**
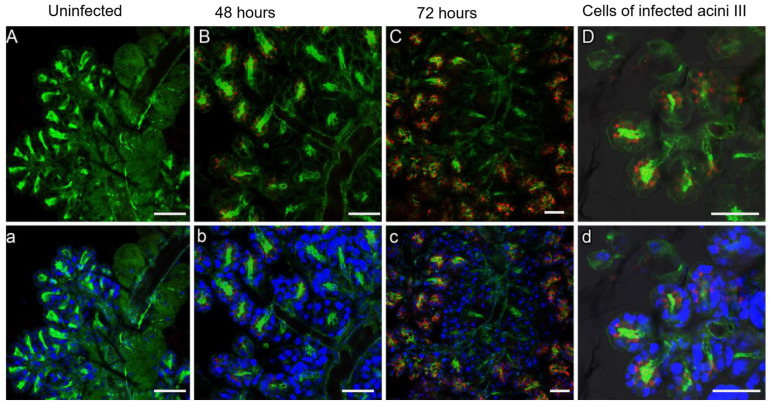
LGTV infection in the salivary glands of blood-feeding female *I. scapularis* ticks. Immunofluorescence of uninfected and infected salivary glands at various timepoints stained for LGTV NS3 (red), for polymerized actin, which is abundant in smooth muscle cells (green), and for nuclei (blue). Uppercase and lowercase images show the same specimen without (uppercase) and with (lowercase) nuclear staining. (**A**,**a**) Uninfected salivary glands of female *I. scapularis* fed for 48 h. Acini of type I had a denser phalloidin stain than those of types II and III. (**B**,**b**) Uninfected salivary glands of *I. scapularis* fed for 48 h and isolated acini type II or III were infected. (**C**,**c**) Infected salivary glands at 72 h. Multiple clusters of acini type II and/or III are infected. (**D**,**d**) Enlarged images of (**B**,**b**). In the infected acini, the peripheral cells were infected, whereas no evidence of infection was found in the lobular ducts. The bar represents 100 µM.

Immunofluorescence for three viral intermediates (NS3, E-protein and dsRNA) and phalloidin for smooth muscle cells also provided direct evidence of LGTV infection of the salivary glands of ticks fed on infected blood for 48 h and 72 h (Figure 6). The signal was observed in granule-containing cells of type II and type III acini (Figure 6B,C). There was no evidence of infection in the cells lining the ducts. Within the individual, infected acinus, the lobular duct cells appeared uninfected; however, the peripheral acinar cells stained positive (Figure 6D). The number of infected cells increased as the duration of feeding increased as shown in Figure 6. We did not observe infection in acini type I.

### 3.5. Comparison of LGTV Infection in the Midgut and Salivary Glands of Blood-Feeding I. scapularis Ticks at 24 h

**Figure 7:**
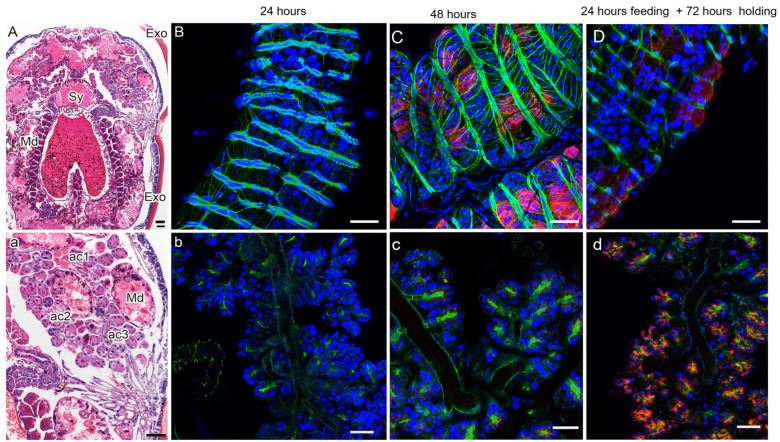
Side-by-side comparison of midgut and salivary glands of infected *I. scapularis* ticks fed for 24 and 48 h and held for 72 additional hours. Uppercase and lowercase images show the midgut and salivary glands from the same tick. (**A**) Histology section stained with H&E stain showing morphological changes in midgut and salivary glands of an adult *I. scapularis* tick. (**a**) Histology section stained with H&E showing the basic structure of salivary glands and different acini. In (**B**–**D**) and (**b**–**d**), the midgut diverticula and the salivary glands were stained for Langat virus (LGTV) dsRNA (red), for polymerized actin, which is abundant in smooth muscle cells (green), and for nuclei (blue). (**B**,**b**) Immunofluorescence of midgut (**B**) and salivary glands (**b**) after 24 h feeding on infected blood. There was no direct evidence of infection in either the midgut or the salivary gland. (**C**,**c**) Immunofluorescence of infected midgut (**C**) and salivary glands (**c**) of a tick feeding on infected blood for 48 h of feeding. The midgut digestive cells and acini type II or III of the salivary glands were targets of infection. (**D**,**d**) Immunofluorescence of infected midgut and salivary glands of a tick feeding on infected blood for 24 h and later held for an additional 72 h. Midgut diverticula (**D**) and the salivary glands (**d**) stained positive for Langat virus (LGTV) dsRNA (red). The bar represents 100 µM.

Direct evidence of infection was not observed when midgut and salivary gland sections were examined 24 h after attachment (Figure 7B,b). However, by 48 h, infection was evident in both the midgut and salivary glands (Figure 7C,c). Because infection might have occurred at 24 h but was below detection limits, we removed the ticks from the chambers at 24 h and held them for an additional 72 h before dissection. In the midgut of ticks that had fed for 24 h and been held for 72 h, virus infection of DG was visible (Figure 7D).

There was no direct evidence of infection in the salivary glands of ticks removed and studied at 24 h. However, if dissected salivary glands were held in culture media for an additional 72 h, infection of type II and type III acini (Figure 7d) was observed. Specifically, if the ticks were removed after 24 h of exposure to infected blood and their dissected organs incubated for an additional 72 h, there was evidence of infection. There was still no evidence of viral infection in the duct network during the study intervals (Figure 7c,d).

### 3.6. Demonstration of Virus Production from the Infected Midguts and Salivary Glands

**Figure 8:**
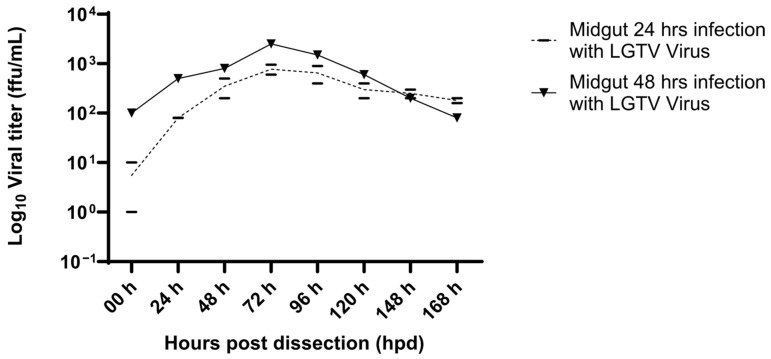
Supernatant from infected ex vivo midgut cultures for 0–168 h after dissection showing viral shedding. The mean viral titer of replicate supernatants at 24 h and 48 h was measured using an immunofocus assay.

Ticks fed on uninfected or LGTV-infected blood for 24 and 48 h were dissected, and the organs then cultured for six days in a Labtek dish with 300 µL L15C-300 medium to assess viral replication and shedding. Immunofocus assay of the supernatants sampled daily during the ex vivo culture showed an almost 2log_10_ increase in infectious virus (Figure 8), further suggesting that LGTV infection occurs within the first 24 h of the start of a blood meal. On the other hand, viral shedding in the salivary glands in the early hours after dissection was similar to that observed in the midgut, with a steady increase until 2 log_10_.

## 4. Discussion

Tick vectors competent to transmit TBFV acquire the virus during a blood meal and subsequently transmit the virus in the next feeding through salivary gland secretions [20]. However, there is limited data on the biology of TBFV infection from the time the arthropod hosts acquire the virus until they transmit it. We used a controlled artificial membrane blood-feeding system and BSL-2 LGTV as a surrogate for Powassan/deer tick virus and other TBFV strains requiring higher levels of containment [31] to elucidate infection and dissemination in ticks.

The midgut is generally assumed to be the initial site of TBFV infection because the virus is delivered via a blood meal, as are other pathogens [20,21,31,32,33]. We identified cells in the digestive cell lineage as targets for virus infection in the midgut (Figure 5 and Figure 7). By 48 h after infection, the virus could be directly identified in generative cells (GCs), digestive cells (DGs), and detached digestive cells (DDGs). However, we also demonstrated incipient infection at 24 h (Figure 7). The number of infected cells increased progressively and there was no evidence of virus infection in cells of the smooth muscle cell network (stained with phalloidin). In our earlier ex vivo studies [21], we observed smooth muscle infection, likely because the entire midgut was dissected out and bathed in virus [22]. These results strongly suggest that cells of the digestive cell lineage were the viral targets and that infection occurs as early as 24 h after attachment.

Identifying LGTV target cells in the salivary gland was more involved. The salivary glands of adult *I. scapularis* comprise several types of acini [33] that are involved either in osmoregulation or production and secretion of the saliva. The acini are connected to a ramifying duct system through which saliva flows to the salivary duct and ultimately exits through the hypostome. The classification of acini is based on morphology as well as the position in the salivary duct tree and the types of granules that the acinar cells contain [34].

When the adult *I. scapularis* ticks were infected with LGTV, viral intermediates were identified in salivary glands by immunofluorescence in granule-containing cells (type II and II acini) at 48 and 72 h post-attachment, consistent with our previous findings from ex vivo cultures [22]. As in the midgut, incipient infection could be demonstrated in ticks removed 24 h after attachment and held an additional 72 h before analysis. Thus, infection of the salivary glands had also likely occurred by 24 h.

Our study has demonstrated that the midgut and salivary glands of adult female ticks are infected within 24 h of attachment. For this to happen, the ingested virus infects the tick midgut, where it rapidly replicates before disseminating to other tissues within the vector, 24 h after ingestion. Our studies revealed a steady increase by 2 logs_10_ in viral shedding from the midgut ex vivo cultures 72 h post-dissection, similar to our previous study [22]. Blood digestion putatively starts in the midgut soon after ingestion, as we observed excretion of tick frass within 24 h of attachment, indicating processing and digestion of the blood meal. This has been the commonly held view that the midgut is infected first, and the virus disseminates from there to other sites, like the salivary glands [17,31]. This traditional view [20,21] is based on results from tick-borne bacterial infections such as Borrelia species, protozoans [32,33,35] and virus infection of insect vectors [20,21]; however, data on viral infections in ticks, particularly the time required to infect a blood-feeding tick, remain limited. The findings leave several unanswered questions. First, by what mechanism does TBFV overcome the midgut host barriers, and how long does this take? Second, does the virus enter the salivary glands directly as the infected blood meal proceeds or by some other less obvious mechanism?

These findings shed light not only on the commonly held view that the midgut is infected first but also on the rapid spread of the virus to the salivary glands. We cannot rule out that some ingested virions may be disseminated without undergoing replication in midgut digestive cells. During intracellular digestion, midgut digestive cells endocytose blood components together with viable virions, and digestion products, including infectious virus, may be released into the hemocoel and rapidly infect the salivary glands. Similarly, there could be a non-midgut entry point via the salivary ducts to the salivary glands, or other organs, such as the synganglion, may play a role in the dissemination.

These findings suggest that the salivary glands of ticks feeding on TBFV-infected blood meal can rapidly become infected while feeding and may subsequently release the virus back into the blood, potentially contributing to amplification and reinfection. This process may affect transmission in male and nymphal ticks.

## 5. Conclusions

In conclusion, our study characterized LGTV infection in the midgut and salivary glands of adult female Ixodes scapularis ticks, demonstrating that it occurs within the first 24 h of an infected blood meal. The targets for viral replication in the midgut were digestive cells of the digestive cell lineage, whereas in the salivary glands, the granule-containing cells in acini types II and III were the target cells.

We examined a single phase of the complex biological process of TBFV infection; there are a number of other crucial questions that remain unanswered: Does a similar sequence and tempo of infection occur in the larval and nymph stages? Does the same pattern of infection occur in adult male ticks? What is the source of the virus that is present in infected larvae, and is it delivered from the male, the female, or both? Is there a role for the synganglion or other organs? Finally, where does the virus persist during molting, when the salivary glands involute and many of the other internal structures degenerate? All these questions should be addressed to gain a complete understanding of the TBFV virus’s biology in the arthropod host.

## Figures and Tables

**Figure A1:**
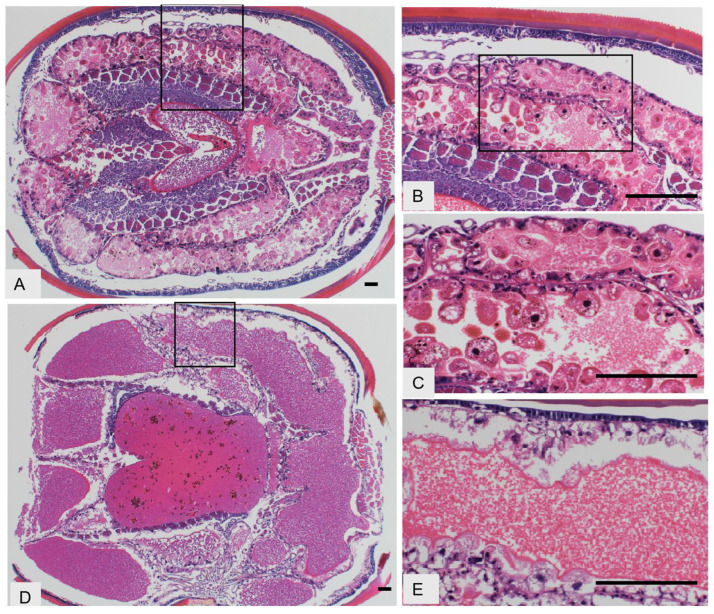
Histological section of midgut of a partially fed and fully fed female *I. scapularis* tick stained with H&E. (**A**) Internal organs of a partially fed tick showing enlargement of the rectal sac and midgut during feeding. (**B**,**C**) Higher-magnification images of (**A**), highlighting detached cells observed within the lumen. (**D**) As feeding continues to 264 h, the midgut and rectal sac enlarge and progressively displace other internal organs. (**E**) Higher magnification of image (**D**) showing the midgut of a fully fed tick, which has mostly a thin stretched cell membrane, and the numerous digestive cells are not as conspicuous as in a partially fed tick. The bar represents 100 µM.

**Figure A2:**
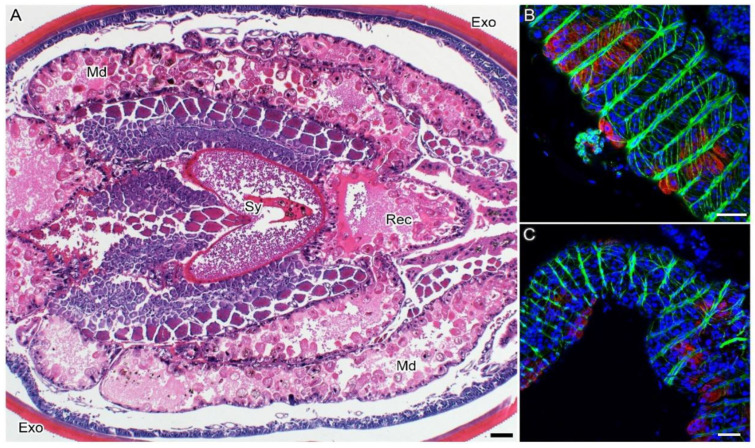
(**A**) Histological section of midgut of a partially fed female *I. scapularis* tick stained with H&E compared to immunofluorescent stains of the midgut (**B**,**C**) to show the morphology and location of the midguts. (**B**,**C**) Clustered infected cells at the later timepoint of 72 h compared to solitary infected cells at early timepoints (Figure 5A–C), the midgut was stained for Langat virus (LGTV) dsRNA (red), for polymerized actin, which is abundant in smooth muscle cells (green), and for nuclei (blue). There was no evidence of viral infection in cells of the smooth muscle cell network (**B**,**C**). The bar represents 100 µM.

## Data Availability

The datasets generated and presented in this article are readily available upon request directed to the corresponding author.

## Abbreviations

The following abbreviations are used in this manuscript:

NIAID	National Institute of Allergy and Infectious Diseases
BSL3	Biosafety Level 3
dsRNA	Double-stranded RNA
DPBS	Dulbecco’s phosphate-buffered saline
DMEM	Dulbecco’s Modified Eagle Medium
E proteins	Envelop Protein
H&E	hematoxylin and eosin stain
LGTV	Langat virus
NS3	Non-Structural protein 3
POWV/DTV	Powassan/deer tick virus
PAS	periodic acid–Schiff stain
TBEV	tick-borne encephalitis virus
TBFV	tick-borne flaviviruses
